# *Actinomyces israelii* May Produce Vulvar Lesions Suspicious for Malignancy

**DOI:** 10.1155/IDOG/2006/48269

**Published:** 2006

**Authors:** Jennifer Y. McElroy, Marsha E. Gorens, Lisa N. Jackson, Danielle Stigger, Teresa Becker, Eyal Sheiner

**Affiliations:** Department of Obstetrics and Gynecology, Rush University Medical Center, Chicago, IL 60612, USA

## Abstract

*Background*. We present a case of *Actinomyces israelii* causing vulvar mass suspicious for malignancy in a postmenopausal woman. *Case*. A 60 year-old woman presented due to a firm, nonmobile, 10 cm vulvar mass, which had been rapidly enlarging for 5 months. The mass was painful, with localized pruritus and sinus tracts oozing of serosanguinous fluid. Biopsy and cultures revealed a ruptured epidermal inclusion cyst containing granulation tissue and *Actinomyces israelii*. *Conclusion*. *Actinomyces israelii* may produce vulvar lesions that are suspicious for malignancy. Thus, biopsies and cultures are both mandatory while evaluating vulvar masses suspicious for malignancy.

## INTRODUCTION

*Actinomyces* is a gram positive, non-spore-forming anaerobic or microaerophilic bacterial rod [1]. *Actinomyces israelii* causes most *Actinomyces* infections in humans, although other forms such as *Actinomyces* Odontolyticus, *Actinomyces* Viscosus, *Actinomyces* Meyeri, *Actinomyces* Gerencseriae, and Propionibacterium Propionicum have also been reported. *Actinomyces* infections are commonly polymicrobial [1]. *Actinomyces israelii* is a normal component of oral and genital tract flora [2, 3] with infections being reported in the oral-cervicofacial, thoracic, abdominal, pelvic, CNS, musculoskeletal regions as well as disseminated disease. The present case describes a postmenopausal woman, without an IUD, presented with a vulvar mass suspicious for neoplasm that was caused by *Actinomyces israelii*.

## CASE REPORT

A 60 year-old, African American woman, gravida 1, para 1, presented to her primary gynecologist with complaints of an enlarging, tender vulvar mass. The patient had first noticed a “lump” 5 months earlier; however she did not seek medical attention until the mass had grown to about 10 cm size. The patient was referred to the hospital for further evaluation, with the suspicious of malignancy.

The patient's past medical history was significant for polyarthritis, and recently diagnosed hypertension well controlled without antihypertensives. She had a hernia surgically repaired in 1998, and had undergone one spontaneous vaginal delivery without complication. She had an unremarkable gynecologic history including a normal pap smear within the last year; no IUD was used since she experienced menopause at 53 years old. She denied hormone or tobacco use. Her family history was negative for breast, ovarian, or colon cancer.

**Figure 1:**
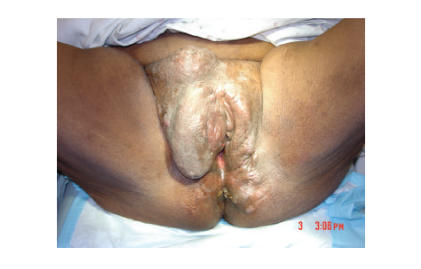
Nodular, firm, nonmobile vulvar mass extending from the right labia majora.

**Figure 2:**
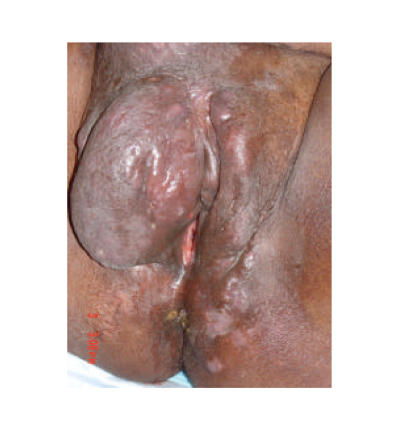
Erythema and draining sinus tract at the superior aspect of the right labial mass.

The patient underwent further evaluation upon admission to the hospital. Examination revealed a 10 cm vulvar mass involving the right mons and right labia majora (Figure 1). The mass was nodular in contour, fixed, nonulcerated, with multiple sinus tracts draining serosanguinous fluid (Figure 2).

**Figure 3:**
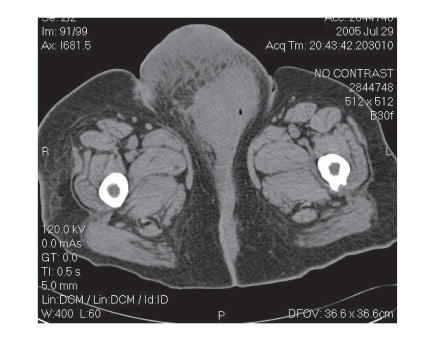
Pelvic CT demonstrating a 10 cm vulvar mass with solid and cystic components, without associated adenopathy.

The cervix was small and mobile as was the uterus on pelvic exam. There were palpable right inguinal nodes. Laboratory evaluation was normal except for an elevated WBC, for which the patient was placed on a broad spectrum antibiotic. A CT of the chest/abdomen/pelvis revealed a normal chest and abdomen. The pelvic CT revealed a 10 cm vulvar mass with solid and cystic components without associated adenopathy (Figure 3).

The patient was subsequently taken to the operating room for vulvar biopsy; a wedge shape tissue sample was taken from the mons lesion, with a smaller tissue samples from the right labia majora and inguinal region. Aerobic and anaerobic cultures were also obtained in the operating room. Pathology revealed a ruptured epidermal inclusion cyst containing granulation tissue, acute and chronic inflammation, and multinucleated giant cells. Cultures were positive for *Actinomyces israelii*, *Propionibacterium acnes*, and *Peptostreptococcus*.

Infectious disease was consulted postoperatively; recommendations included an intravenous course of third generation cephalosporin for 6 weeks, followed by oral antibiotics until disease resolution is observed. Three options were discussed with the patient: expectant management, medical therapy, or surgical extirpation followed by antibiotics. The patient opted for antibiotic treatment. A PICC line was placed to facilitate extended IV antibiotic administration of ceftriaxone (Rocephin; 2 gm IV for 12 weeks). Home health nursing was arranged, and the patient was discharged home in stable condition.

## DISCUSSION

Our case illustrates an atypical presentation of *Actinomyces israelii* as a large vulvar mass in a postmenopausal patient. *Actinomyces israelii* of the vulva is a rare occurrence, represented by limited reports [4]. It is thought that infection occurs after disruption of the mucosal barrier. The lesion appearance is a single or multiple indurated masses, which may contain flocculent areas; these lesions are often mistaken for neoplasms. Progression is slow, often with development of draining sinus tracts.

Diagnosis of *Actinomyces israelii* is difficult, as even one dose of antibiotics prior to culture can obscure results [1]. Culture requires 5–7 days but may take 2–4 weeks. “Sulfur granules” are actually yellow colored aggregates of microorganisms; they do not contain sulfur and are therefore a misnomer. These are usually isolated from purulent material and can be visible macroscopically as well as microscopically. Not all *Actinomyces* species form sulfur granules. Treatment classically begins with IV penicillin for 2–6 weeks, followed by oral therapy with pencillin or amoxicillin for 6–12 months. For penicillin allergic patients, tetracycline, erythromycin, minocycline and clindamycin have been administered. Imipenem and ceftriaxone have been described as successful in reports. Medical therapy is an acceptable treatment option if the patient is stable and reliable for follow up. If, however, the patient is critical or the disease site is critical, a combined approach may be more reasonable.

Pelvic infections involving *Actinomyces* in association with the use of IUDs are well established [5, 6]. Since our patient was postmenopausal, with no evidence of IUD, *Actinomyces israelii* was not suspected.

In conclusion, *Actinomyces israelii* may produce vulvar lesions that are suspicious for malignancy. Thus, biopsies and cultures are both mandatory while evaluating vulvar masses suspicious for malignancy.
